# Joint User Association, Power Allocation and Beamforming for NOMA-Based Integrated Satellite–Terrestrial Networks

**DOI:** 10.3390/e26121055

**Published:** 2024-12-05

**Authors:** Peizhe Xin, Zihao Fu, Zhiyi Chen, Jing Jiang, Jing Zou, Yu Zhang, Xinyue Hu

**Affiliations:** 1 State Grid Economic and Technological Research Institute Co., Ltd., 102209 Beijing, China; xinpeizhe@chinasperi.sgcc.com.cn (P.X.); 18810555907@163.com (Z.F.); chenzhiyi@chinasperi.sgcc.com.cn (Z.C.); jiangjing@chinasperi.sgcc.com.cn (J.J.); zoujing@chinasperi.sgcc.com.cn (J.Z.); 2 Information Materials and Intelligent Sensing Laboratory of Anhui Province, Anhui University, Hefei 230601, China; xinyuehu@ahu.edu.cn

**Keywords:** integrated satellite–terrestrial network (ISTN), non-orthogonal multiple access (NOMA), user association, power allocation, beamforming

## Abstract

This paper investigated a non-orthogonal multiple access (NOMA)-based integrated satellite–terrestrial network (ISTN), where each user can select to access a terrestrial base station (BS) or the satellite according to the capacity of BS and their individual transmission requirements. A two-stage algorithm is proposed to solve the achievable sum rate maximizing resource optimization problem. In the first stage, user associations are determined based on individual preference lists and the backhaul capacities of the access points (APs). In the second stage, the power allocation, and the receiving beamforming vectors are optimized alternately. Within each iteration, the closed-form solution for the transmit power is derived. Simulation results show the effectiveness of the proposed algorithm and the benefits brought by NOMA. When the backhaul link capacity of terrestrial BSs is sufficient, users (UEs) prefer to access these BSs. Otherwise, the satellite can offer QoS guarantees to UEs. Furthermore, the overall system performance reaches its optimum when the number of UEs in the system matches the number of receive antennas at the APs.

## 1. Introduction

Although terrestrial cellular networks undergo several generations of evolution, half of the world still faces the challenges of achieving ubiquitous access, primarily due to the economic costs associated with base station (BS) deployment [1,2]. To address this challenge, the third-generation partnership project (3GPP) promotes a promising architecture, referred to as integrated satellite–terrestrial networks (ISTNs) [3,4], which integrate satellite communication and terrestrial networks and can provide wide-area coverage, universal multiaccess, and ubiquitous connectivity. ISTNs have a wide range of applications, such as personal mobile communication, transportation, aviation and navigation, Power Internet of Things (PIoT), emergency disaster relief, and so on.

Despite the advantages of ISTNs, scarce frequency resource and increased interference caused by heterogeneous networks limit their transmission performance. To overcome this, many researchers devote their efforts to studying resource allocations in ISTNs, mainly focusing on user scheduling, power allocation, and beamforming [5,6,7,8]. In [5], a distributed user association with a grouping mechanism was proposed to maximize the sum rate and balance the load of ISTN by jointly considering the backhaul capacity of BSs and mobility and delay of users (UEs). In [6], a  power and frequency resource allocation scheme was proposed to minimize total energy and maximize spectral efficiency. A power control scheme for cognitive ISTNs was proposed in [7], in which the aim is to maximize the energy efficiency subject to interference power constraints imposed by terrestrial communications and outage constraints of satellite communications. In [8], the authors studied hybrid beamforming, user scheduling, and power allocation optimization in ISTN with the purpose of improving system sum rate and energy efficiency. The authors in  [9] considered an uplink communication model consisting of a single satellite, a single terrestrial station, and multiple ground users. In this model, the  BS serves as a satellite–terrestrial relay, bridging users and the satellite. The  sum rate was maximized by optimizing user grouping and transmit power of users. Although elaborate resource allocation design can mitigate interference effectively, with the surge in user numbers, the congestion of spectrum resources has seriously degraded the communication rate in ISTNs.

Recently, non-orthogonal multiple access (NOMA) technology has presented an effective solution to promote spectrum efficiency. In a NOMA system, multiple UEs can share the same spectrum resource, with interference being effectively mitigated by the adoption of successive interference cancellation (SIC) technology at the receiver [10,11]. NOMA has been widely investigated in terrestrial networks. Ref. [12] investigated a sum rate maximization problem in a movable antenna-enabled uplink NOMA system. The author proposed a successive convex approximation-based algorithm to jointly optimize the movable antennas’ positions, the decoding order, and the power control. The authors in [13] formulated a joint power allocation and channel assignment problem for uplink NOMA-based wireless communication networks. In order to solve the problem, a methodology that integrates convex optimization and machine learning techniques was proposed. In [14], a resource allocation problem for the coexistence of enhanced mobile broadband (eMBB) and ultra-reliable low-latency communication (URLLC) traffic scheduling was investigated; the sum rate of all eMBB users was maximized while maintaining the minimum data rate requirement of each eMBB user.

Research reveals that NOMA offers significant performance advantages compared with the traditional orthogonal multiple access (OMA) schemes. The integration of NOMA into ISTNs to enhance communication performance and resource utilization tends to be an inevitable trend. Considering applying NOMA technology to terrestrial networks, [1] and [15] investigated downlink ISTNs. The authors in [15] studied user association, user pairing, subchannel allocation, and power allocation algorithms. The work in [1] proposed a reconfigurable intelligent surface (RIS)-assisted downlink ISTN with NOMA, in which the energy efficiency was maximized by jointly optimizing the active beamforming vector at the BS and the passive beamforming matrix at the RIS. In [2], the authors investigated the joint optimization algorithm for the phase shift, transmit power, and receive beamforming of an uplink RIS-assisted NOMA-based ISTN. In the considered scenario, only one BS is deployed, and the direct UEs are served by space division multiple access technology. The authors of  [16] investigated an uplink caching-based NOMA ISTN, where the satellite provides backhaul links for terrestrial BSs. In this work, a system utility function, which consists of the achieved terrestrial user rate and cross-tier interference caused by terrestrial BSs to satellite, was maximized by jointly optimizing user association, bandwidth assignment, and power allocation. In [17], a satellite–aerial–terrestrial uplink network was investigated. The authors considered a high-altitude platform as a grant-based entity serving multiple mobile terminals to realize satellite–terrestrial uplink communication. NOMA was also applied on the high-altitude platform to increase communication capacity.

Although many studies have focused on resource allocation in NOMA-based ISTNs, a majority of these papers have only implemented NOMA for a portion of UEs. And joint resource optimization is mainly divided into two categories: One considers fixed user association, optimizing power, and beamforming, which means UEs cannot flexibly choose to access BSs or satellites according to their individual requirements. The other category allows UEs to choose to access BSs or satellites, optimizing user association and power allocation. However, such research often assumes that all communication nodes are equipped with a single antenna, and therefore does not need to consider the impact of beamforming on user association. The joint optimization of user association, beamforming, and power allocation in NOMA-based ISTNs still poses challenges. Motivated by this, we investigated joint resource optimization in an uplink NOMA-based ISTN, where NOMA is applied for all UEs and each user can choose to access either the satellite or terrestrial BSs based on their communication rate requirements. Note that this work differs significantly from the investigations in [2,9,16,17], both in terms of the scenarios studied and the parameters optimized. The main contributions are summarized as follows:

- We propose an uplink NOMA-based ISTN, where each user can access either BSs or the satellite. With the goal of maximizing the achievable sum rate of all the UEs, an optimization problem of jointly optimizing the transmit power, receiving beamforming, and user association is established by considering the backhaul link capacity of BSs and the satellite, as well as individual quality of service (QoS) constraints.
- We develop a two-stage algorithm to solve the problem. At the first stage, each user is associated with a corresponding access point (AP), i.e., either a BS or the satellite, based on their preference list and the backhaul link capacity of the APs. At the second stage, the power allocation and the receiving beamforming vectors are optimized iteratively. Within each iteration, the closed-form solution for the transmit power is derived.
- The simulation results demonstrate the superiority of the proposed scheme compared with the random power allocation scheme and the traditional OMA scheme. In scenarios where the backhaul link capacity of terrestrial BSs is sufficient, UEs tend to access these BSs. However, when the backhaul link capacity of terrestrial BSs is insufficient, the satellite can offer QoS guarantees to UEs. Moreover, the system’s overall performance achieves its peak when the number of UEs in the system aligns with the number of receive antennas at the APs.

## 2. System Model and Problem Formulation

### 2.1. System Model

**Figure 1:**
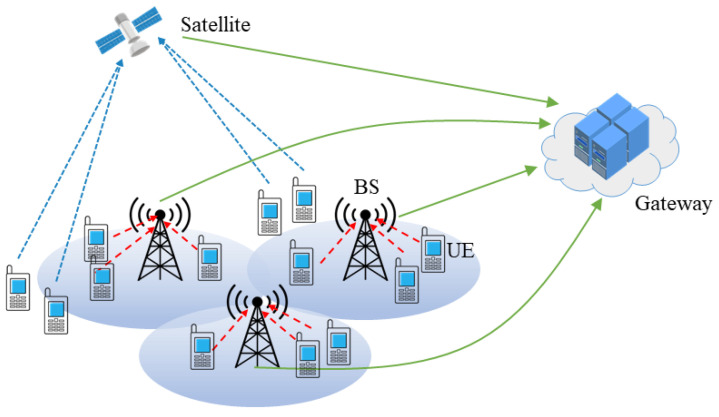
The NOMA-based terrestrial–satellite network architecture.

As shown in Figure 1, an uplink communication scenario for the terrestrial–satellite network is investigated, in which *M* BSs and one satellite (The proposed algorithm can be easily extended to multiple satellite settings. The additional satellites can be viewed as additional APs.) are deployed to serve *K* terrestrial UEs. The UEs’ data is uploaded to the BSs or satellite and then transmitted to the gateway of the core network via the backhaul links. Define the sets of UEs and BSs as K={1,2,…,K} and M={1,2,…,M}, respectively. The satellite is denoted as M+1. And all the APs (i.e., BSs and satellite) constitute a set denoted by R=M∪M+1. Assume that the *m*-th AP is equipped with $N_{m}$, m=1,…,M+1 antennas, while the terrestrial UEs are all deployed with single antennas. Denote $U_{m}$ as the UEs associated to AP *m*, satisfying $K=\{U_{1}\cup U_{2}\ldots \cup U_{M+1}\}$, then the received signal of AP *m* from UE *k* is denoted by

(1) $$y_{m,k}=h_{m,k}\sqrt{p_{m,k}}s_{m,k}+\sum_{j\in U_{m}\setminus k}h_{m,j}\sqrt{p_{m,j}}s_{m,j}+\sum_{i=1,i\neq m}^{M+1}\sum_{j\in U_{i}}h_{m,j}\sqrt{p_{i,j}}s_{i,j}+n_{m},$$

where $p_{m,k}$ and $s_{m,k}$ denote the transmit power and symbol of UE *k* served by AP *m*. $h_{m,k}\in C^{N_{m}\times 1}$ denotes the channel vector of UE *k* towards AP *m*. $n_{m}∼\mathrm{CN}\left(0,\sigma_{m}^{2}I\right)$ is the additive white Gaussian noise (AWGN) vector. Similar to most of the related works [4,18], we assume that all the wireless channels undergo slow fading, and the perfect channel state information of all channels are available at the gateway through feedback/training sent from the user terminals via the backhaul channel. This mechanism has already been adopted in DVB-S2 [19]. For the uplink NOMA communication scenario, the UEs with better channel conditions have the higher priority to be decoded through SIC [2]. Assume that channel coefficients of UEs towards AP *m* are sorted as

(2) $$Q_{m}≜{\left|h_{m,\pi_{m}\left(1\right)}\right|}^{2}\geq {\left|h_{m,\pi_{m}\left(2\right)}\right|}^{2}\geq \cdots \geq {\left|h_{m,\pi_{m}\left(K\right)}\right|}^{2},\forall m\in R.$$

Let $S_{m,k}=\left\{\pi_{m}\left(1\right),\pi_{m}\left(2\right),\cdots ,\pi_{m}\left(k\right)\right\}$; thus, the received SINR of UE *k* at AP *m* is calculated by

(3) $$SINR_{m,k}=\frac{{\left|v_{m,k}h_{m,k}\right|}^{2}p_{m,k}}{\sum_{j\in U_{m}\setminus S_{m,k}}{\left|v_{m,k}h_{m,j}\right|}^{2}p_{m,j}+\sum_{i=1,i\neq m}^{M+1}\sum_{j\in U_{i}}{\left|v_{m,k}h_{m,j}\right|}^{2}p_{i,j}+{∥v_{m,k}∥}^{2}\sigma_{m}^{2}},$$

where $v_{m,k}\in C^{1\times N_{m}}$ is the receive beamformer for AP *m* to detect the signal from UE *k*. Thus, the achievable rate of UE *k* at AP *m* is given by

(4) $$R_{m,k}=\log\left(1+SINR_{m,k}\right)$$

Define $\alpha_{m,k}$ as the UE association indicator, where $\alpha_{m,k}=1$ denotes that UE *k* is served by AP *m*, and $\alpha_{m,k}=0$, otherwise. Therefore, the achievable transmission rate of UE *k* is written as

(5) $$R_{k}=\sum_{m\in R}\alpha_{m,k}R_{m,k}$$

And the achievable sum rate for AP *m* is given by

(6) $$R_{m}=\sum_{k\in K}\alpha_{m,k}R_{m,k}$$

### 2.2. Problem Formulation

In this work, we aim to maximize the achievable sum rate of all terrestrial UEs by optimizing the transmit power, receive beamformer, and UE association. The proposed problem is formulated as

(7) maxvm,k,pm,k,αm,kRsum=∑k∈KRks.t.C1:pm,k≤Pkmax,∀k∈K,m∈R,s.t.C2:αm,k∈0,1,∀k,m,s.t.C3:∑m∈Rαm,k=1,∀k,s.t.C4:Rm≤Cm,∀m∈M,s.t.C5:Rk≥rk,∀k,

where C1 limits the maximum transmit power of UE *k*, C2 constrains the UE user association indicators, and C3 denotes that a UE can only access one AP. Constraint C4 denotes the achieved sum rate of BS *m* should be less than its backhaul link capacity $C_{m}$. Constraint C5 guarantees the UE’s QoS. Note that this work primarily considers communication scenarios where the access capacity of terrestrial BSs is limited, necessitating the introduction of satellites to provide service for terminals unable to connect to BSs. Consequently, satellites are specifically employed to guarantee normal communication in this region, and typically, satellite downlink communication rates are high. Thus, the backhaul link capacity constraint is not imposed on the satellite.

The optimization problem (7) is a mixed-integer non-linear programming problem, characterized by its pronounced non-convexity and NP complexity. Additionally, the update of the user association strategy triggers alterations in user channel conditions, making the direct transformation of the optimization problem (7) into a convex form exceedingly difficult. To address this complexity in a more efficient manner, the problem (7) has been strategically partitioned into three stages for resolution.

## 3. The Proposed Optimization Algorithm

### 3.1. User Association

With the fixed receive beamforming vector $v_{m,k}$ and transmit power $p_{m,k}$, problem (7) is reduced to the following form

(8) $$\begin{array}{c} \max_{\alpha_{m,k}}R_{sum}=\sum_{k\in K}R_{k}\\ s.t.\;C2,C3,C4,C5, \end{array}$$

which is a non-convex integer programming that is difficult to solve. Here, we temporarily ignore the QoS constraint C5 and design an approach to determine user association.

Preparation: All the UEs establish their preference lists $\left\{Pre_{k}\right\}$ in accordance with the descending order of the channel gain towards the APs.

Judge and Join-in: Define $X_{1}$ as the set of UEs that have already accessed an AP and  $X_{2}$ as the set of UEs that have not yet accessed any AP. Each UE in $X_{2}$ sequentially sends the request signal to the APs in order of priority based on $\left\{Pre_{k}\right\}$. The  AP determines whether the inclusion of the current user would cause the sum rate to exceed the backhaul link rate. If not, the AP allows the current user access. This judgment step is to ensure that the constraint C4 in (8) is satisfied. However, a challenging problem is that the subsequent UE association strategy, receive beamforming design, and power allocation can all affect the achievable sum rate of the current AP. To address this, an approximate upper bound on the achievable sum rate is derived as a criterion for making the decision.

Let $\alpha_{m}={\left[\alpha_{m,1},\alpha_{m,2},\ldots ,\alpha_{m,K}\right]}^{T}$ and define $H_{m}=\left[h_{m,1},\ldots ,h_{m,K}\right]\mathrm{diag}\left(\alpha_{m}\right)$ as the channel matrix from the UEs served by AP *m* to AP *m*, where diag(α) represents a diagonal matrix constructed from the vector α. Denote the interference channel matrix from the UEs served by AP *i*, i∈R∖m to AP *m* by

(9) $${\bar{H}}_{m}=\left[H_{1}\mathrm{diag}\left(\alpha_{1}\right),\ldots ,H_{m-1}\mathrm{diag}\left(\alpha_{m-1}\right),H_{m+1}\mathrm{diag}\left(\alpha_{m+1}\right)\ldots ,H_{M+1}\mathrm{diag}\left(\alpha_{M+1}\right)\right],$$

then, according to the minimum mean square error (MMSE) criterion, the upper bound of the achievable sum rate for AP *m* is

(10) $$R_{m}^{up}=\log\det\left(I+\frac{H_{m}{\mathrm{PH}}_{m}^{H}}{{\bar{H}}_{m}P{\bar{H}}_{m}^{H}+\sigma_{0}^{2}I}\right),$$

where $P=\mathrm{diag}\left(p\right)=\mathrm{diag}\left({\left[p_{1},p_{2},\ldots ,p_{K}\right]}^{T}\right)$ and $p_{k}=\sum_{m\in R}\alpha_{m,k}p_{m,k}$. The value of $p_{m,k}$ is set to the maximum, and  $\alpha_{m,k}$ for $k\in X_{2}$ is set to 0.

After the last AP reaches its saturation rate, if $X_{2}\neq \emptyset$, the remaining UEs reset their preference lists and repeat the above process.

### 3.2. Power Allocation

With the fixed receive beamforming vector $v_{m,k}$ and UE association indicator $\alpha_{m,k}$, problem (7) is reduced to the following form:

(11) maxpm,k∑m∈R∑k∈UmRm,ks.t.C1′:pm,k≤Pkmax,∀m∈R,∀k∈Um,s.t.C4′:∑k∈UmRm,k≤Cm,∀m∈M,s.t.C5′:Rm,k≥rk,∀m∈R,∀k∈Um,

By introducing the Lagrange multiplier $\rho_{k}$ associated with the constraint C5′, the partial Lagrange function of problem (11) can be derived as

(12) $$\begin{array}{c} \max_{p_{m,k}}\sum_{m\in R}\sum_{k\in U_{m}}R_{m,k}+\sum_{m\in R}\sum_{k\in U_{m}}\rho_{k}\left(R_{m,k}-r_{k}\right)\\ s.t.\;C1^{\prime },{C4}^{\prime } \end{array}$$

To tackle the non-convexity of (12), we first introduce the quadratic transform method in [20] to convert log-fractional rate functions in the objective function into convex quadratic expressions. By introducing auxiliary variables $\gamma_{m,k}$ and $\eta_{m,k}$, the log-fractional rate function can be transformed into

(13) $$\begin{array}{cc} {\tilde{R}}_{m,k}≜ & \log\left(1+\gamma_{m,k}\right)-\gamma_{m,k}+2\eta_{m,k}\sqrt{\left(1+\gamma_{m,k}\right){\left|v_{m,k}h_{m,k}\right|}^{2}p_{m,k}}\\ \; & -\eta_{m,k}^{2}\left(\sum_{j\in \left\{U_{m}\setminus S_{m,k}\right\}\cup k}{\left|v_{m,k}h_{m,j}\right|}^{2}p_{m,j}+\sum_{i\in R,i\neq m}\sum_{j\in U_{i}}{\left|v_{m,k}h_{i,j}\right|}^{2}p_{i,j}+{∥v_{m,k}∥}^{2}\sigma_{m}^{2}\right) \end{array}$$

By substituting ${\tilde{R}}_{m,k}$ into (12), the problem is then transformed into the following:

(14) $$\begin{array}{c} \max_{p_{m,k}}\sum_{m\in R}\sum_{k\in U_{m}}{\tilde{R}}_{m,k}+\sum_{m\in R}\sum_{k\in U_{m}}\rho_{k}\left({\tilde{R}}_{m,k}-r_{k}\right)\\ s.t.\;C1^{\prime },{C4}^{\prime } \end{array}$$

Given $p_{m,k}$, $\gamma_{m,k}$ and $\eta_{m,k}$ can be updated by

(15) $$\gamma_{m,k}=\frac{{\left|v_{m,k}h_{m,k}\right|}^{2}p_{m,k}}{\sum_{j\in U_{m}\setminus S_{m,k}}{\left|v_{m,k}h_{m,j}\right|}^{2}p_{m,j}+\sum_{i\in R,i\neq m}\sum_{j\in U_{i}}{\left|v_{m,k}h_{i,j}\right|}^{2}p_{i,j}+{∥v_{m,k}∥}^{2}\sigma_{m}^{2}}$$

(16) $$\eta_{m,k}=\frac{\sqrt{\left(1+\gamma_{m,k}\right){\left|v_{m,k}h_{m,k}\right|}^{2}p_{m,k}}}{\sum_{j\in \left\{U_{m}\setminus S_{m,k}\right\}\cup k}{\left|v_{m,k}h_{m,j}\right|}^{2}p_{m,j}+\sum_{i\in R,i\neq m}\sum_{j\in U_{i}}{\left|v_{m,k}h_{i,j}\right|}^{2}p_{i,j}+{∥v_{m,k}∥}^{2}\sigma_{m}^{2}}$$

With fixed $\gamma_{m,k}$ and $\eta_{m,k}$, the residual power allocation of (14) is convex without C4′. Accordingly, during the iterative process, we temporarily disregard constraint C4′. Following the completion of the iterations, adjustments will be made to the power allocation in order to satisfy constraint C4′. The corresponding Lagrangian dual function is derived as

(17) $$L\left(p_{m,k}\right)=\sum_{m\in R}\sum_{k\in U_{m}}\left({\tilde{R}}_{m,k}+\rho_{k}\left({\tilde{R}}_{m,k}-r_{k}\right)\right)$$

The Karush-Kuhn-Tucker (KKT) conditions are

(18) $$\begin{array}{cc} \frac{\partial L\left(p_{m,k}\right)}{\partial p_{m,k}}= & \left(1+\rho_{k}\right)\eta_{m,k}\sqrt{\frac{\left(1+\gamma_{m,k}\right){\left|v_{m,k}h_{m,k}\right|}^{2}}{p_{m,k}}}-\sum_{k^{\prime }\in S_{m,k}\cup k}\left(1+\rho_{k^{\prime }}\right)\eta_{m,k^{\prime }}^{2}{\left|v_{m,k^{\prime }}h_{m,k}\right|}^{2}\\ \; & -\sum_{m^{\prime }\in R\setminus m}\sum_{k^{\prime }\in U_{i}}\left(1+\rho_{k^{\prime }}\right)\eta_{m^{\prime },k^{\prime }}^{2}{\left|v_{m^{\prime },k^{\prime }}h_{m,k}\right|}^{2}=0,\;\;\forall m\in R,k\in U_{m}, \end{array}$$

Then, the optimal power allocation is derived by

(19) $$p_{m,k}^{*}=\min\left\{{\tilde{p}}_{m,k},P_{k}^{max}\right\}$$

where

(20) $${\tilde{p}}_{m,k}=\frac{{\left(\left(1+\rho_{k}\right)\eta_{m,k}\right)}^{2}\left(1+\gamma_{m,k}\right){\left|v_{m,k}h_{m,k}\right|}^{2}}{{\left(\sum_{k^{\prime }\in S_{m,k}\cup k}\left(1+\rho_{k^{\prime }}\right)\eta_{m,k^{\prime }}^{2}{\left|v_{m,k^{\prime }}h_{m,k}\right|}^{2}+\sum_{m^{\prime }\in R\setminus m}\sum_{k^{\prime }\in U_{i}}\left(1+\rho_{k^{\prime }}\right)\eta_{m^{\prime },k^{\prime }}^{2}{\left|v_{m^{\prime },k^{\prime }}h_{m,k}\right|}^{2}\right)}^{2}}$$

The Lagrangian multipliers $\rho_{k}$ can be obtained by utilizing the gradient update method [21]. Specifically, in the *t*-th iteration, the Lagrangian multiplier is updated by the following expression

(21) $$\rho_{k}^{\left(t\right)}={\left[\rho_{k}^{\left(t-1\right)}-\phi_{k}\left({\tilde{R}}_{m,k}-r_{k}\right)\right]}^{+},$$

where the update step-size $\phi_{k}$ plays a crucial role in controlling the convergence of the Lagrangian multiplier.

After iterations, we need to deal with constraint C4’. Assume $\tilde{R}$ representing the set of APs that cannot meet $\sum_{k\in U_{m}}R_{m,k}\leq C_{m}$, then we set $\Delta C_{m}=\sum_{k\in U_{m}}R_{m,k}-C_{m}$, $\forall m\in \tilde{R}$. The power allocation results can be adjusted as

(22) $$p_{m,k}=\left(2^{\left(R_{m,k}-\Delta C_{m}\left(R_{m,k}/\sum_{j\in U_{m}}R_{m,j}\;\right)\right)}-1\right)\frac{I_{m,k}}{{\left|v_{m,k}h_{m,k}\right|}^{2}},\;\;\forall m\in \tilde{R}$$

where $I_{m,k}=\sum_{j\in U_{m}\setminus S_{m,k}}{\left|v_{m,k}h_{m,j}\right|}^{2}p_{m,j}+\sum_{i=1,i\neq m}^{M+1}\sum_{j\in U_{i}}{\left|v_{m,k}h_{m,j}\right|}^{2}p_{i,j}+{∥v_{m,k}∥}^{2}\sigma_{m}^{2}$.

### 3.3. Receive Beamforming

With the given transmit power $p_{m,k}$ and UE association indicator $\alpha_{m,k}$, the receive beamforming vectors $v_{m,k}$, ∀m,k, can be obtained based on the optimal MMSE criteria to cope with the multiuser interference, which is obtained by

(23) $$v_{m,k}^{*}=\arg\max SINR_{m,k},$$

which can be further represented as

(24) $$v_{m,k}^{*}=\arg\max\frac{v_{m,k}Q_{1}v_{m,k}^{H}}{v_{m,k}Q_{2}v_{m,k}^{H}},$$

where $Q_{1}=h_{m,k}p_{m,k}h_{m,k}^{H}$, $Q_{2}=\sum_{j\in U_{m}\setminus S_{m,k}}h_{m,j}p_{m,j}h_{m,j}^{H}+\sum_{i=1,i\neq m}^{M+1}\sum_{j\in U_{i}}h_{m,j}p_{i,j}h_{m,j}^{H}+\sigma_{m,k}^{2}I$. It can be seen that (24) is a generalized Rayleigh quotient from [22]. Thus, the optimal solution is the eigenvector corresponding to the maximum generalized eigenvalue of matrix $Q_{2}^{-1}Q_{1}$, which is written as

(25) $$v_{m,k}^{*}=\lambda_{max}\left(Q_{2}^{-1}Q_{1}\right)$$

### 3.4. Complexity Analysis

The proposed two-stage algorithm to solve (7) is summarized in Algorithm 1. The first stage comprises steps 2 to 17, and the second stage comprises steps 18 to 25. In the first stage, the key operation is to calculate the achievable sum rate in (10), which needs a determinant operation with computational complexity $O\left(N!\right)$. Thus, the complexity for the first stage in the worst case is $O\left(N!K\left(M+1\right)T_{1}\right)$. Here, $T_{1}$ is a predefined maximum number of user association rounds. In the second stage, the key operation is to calculate $v_{m,k},\gamma_{m,k},\eta_{m,k}$ and $p_{m,k}$. The complexity to calculate those parameters is $O\left(N^{3}\right)$, $O\left(N\right)$, $O\left(N\right)$ and $O\left(N\right)$, respectively. Thus, the complexity of the second stage is $O\left(\left(T_{2}^{in}(M+1)KN+\left(M+1\right)KN^{3}\right)T_{2}^{out}\right)$. Here, $T_{2}^{out}$ and $T_{2}^{in}$ represent the maximum iteration numbers for the outer and inner iterations, respectively.
**Algorithm 1:** The proposed two-stage algorithm to solve (7)
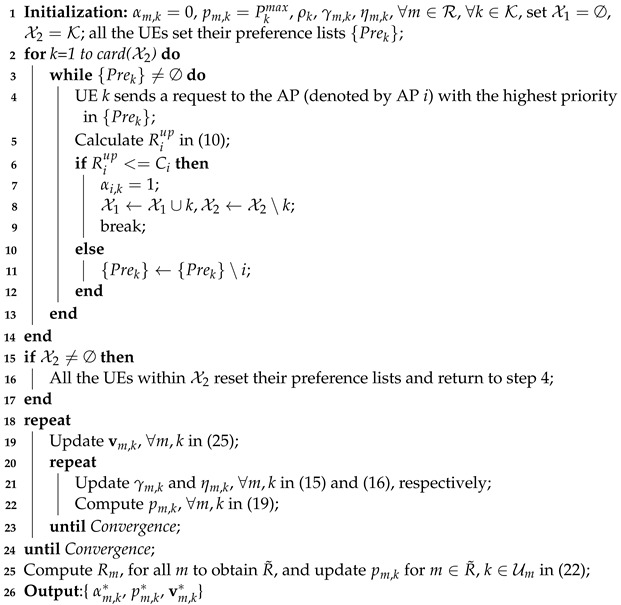


According to the above analysis, the total computational complexity for our proposed algorithm is $O\left(\left(T_{2}^{in}(M+1)KN+\left(M+1\right)KN^{3}\right)T_{2}^{out}+N!K\left(M+1\right)T_{1}\right)$.

## 4. Simulation Results

This section presents simulation results to validate the performance of our proposed algorithms. We consider 4 BSs serving a square region on the ground with a size of 1 km × 1 km. The coordinates of the four vertices of the considered square region are (0 km, 0 km), (1 km, 0 km), (1 km, 1 km), and (0 km, 1 km), respectively. The four BSs are uniformly placed in the square region, with coordinates of (0.25 km, 0.25 km), (0.25 km, 0.75 km), (0.75 km, 0.75 km), and (0.75 km, 0.25 km), respectively. A low Earth orbit (LEO) satellite at an altitude of 300 km is deployed to serve the considered region. All the UEs are evenly and randomly distributed within the considered square region. The channels between UEs and BSs are modeled as Rayleigh channels [23], and the channels between UEs and the satellite are modeled as far-field line-of-sight channels [24]. The path loss exponents for the two types of links are set to 3 and 2, respectively. The maximum transmit powers $P_{k}^{max}$ for all k∈K are set to be the same. Table 1 summarizes the simulation parameters. The simulations were conducted using Matlab 2021 B software, yielding numerical results from Monte Carlo simulations with 100 independent channel realizations.

**Figure 2:**
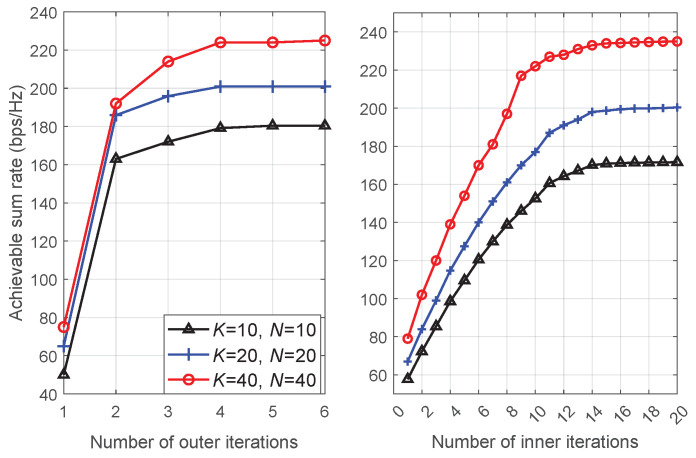
Convergence of the proposed algorithm under different settings of *K* and *N*.

Figure 2 gives the convergence of the proposed algorithm under different settings of *K* and *N*. The main iteration steps occur in the inner and outer iterations of stage 2 in Algorithm 1. During the inner iteration, parameters $\gamma_{m,k}$, $\eta_{m,k}$ are updated. The outer iteration involves the alternative optimization of $v_{m,k}$ and $p_{m,k}$. Consequently, in the revised manuscript, we incorporated the convergence behavior for both the inner and outer iterations of stage 2 in Algorithm 1. It is seen that for both inner and outer iterations, the convergence is monotonic under all settings of *K*, *N*. For the inner iteration, it takes approximately 15 iterations to reach convergence. For the outer iteration, about four iterations are required to converge.

**Figure 3:**
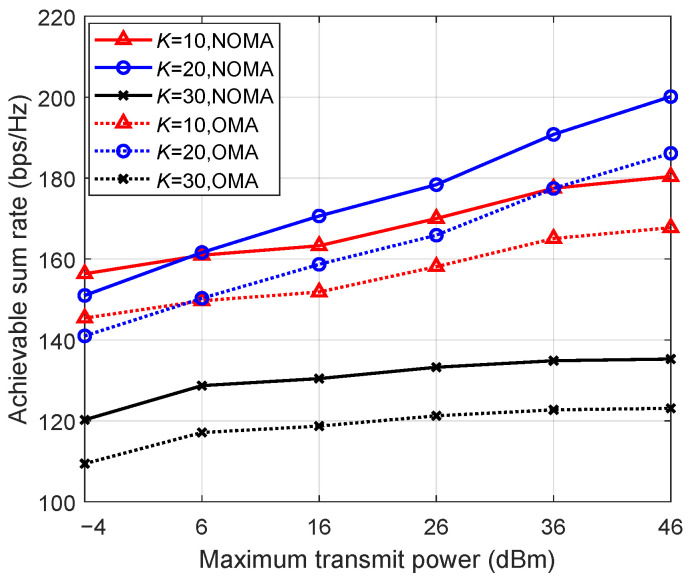
Achievable sum rate versus the maximum transmit power.

Figure 3 presents the achievable sum rate versus the maximum transmit power. The total number of UEs is set to K=10, K=20, K=30. The number of receive antennas is set to N=20. It is seen that the achievable sum rate increases with the maximum transmit power. The NOMA scheme is obviously better than the OMA scheme, which indicates the benefits of adopting NOMA in uplink ISTNs. The performance degrades when the number of UEs is set to 30. This is because, when the number of receive antennas exceeds the number of UEs, the system has a sufficient degree of freedom to distinguish signals from different UEs and minimize interference among them. However, once the number of UEs exceeds the number of antennas, the system lacks the necessary degree of freedom for leveraging multiuser diversity, resulting in the degradation of system performance.

**Figure 4:**
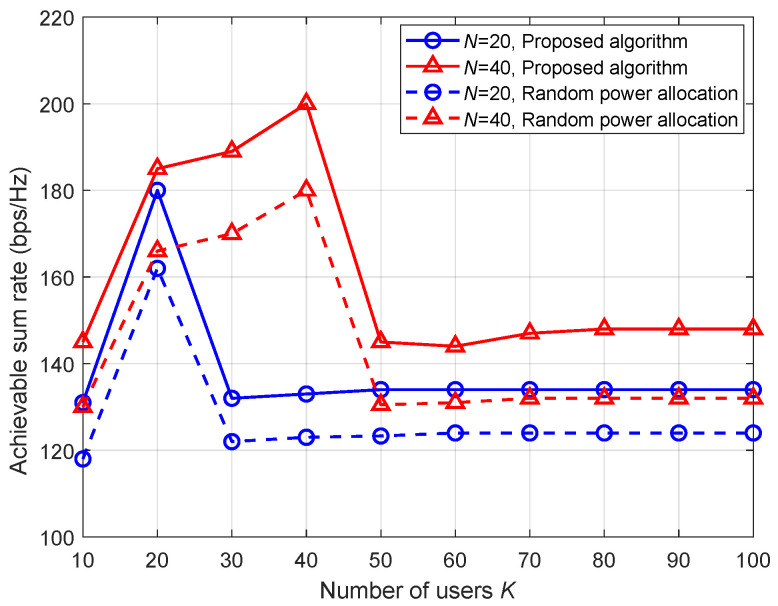
Achievable sum rate versus the number of UEs.

Figure 4 presents the achievable sum rate versus the number of UEs. The maximum transmit power is set as 26 dBm. The numbers of receive antennas for different APs are set to the same constant *N*. It is seen that the achievable sum rate increases with the number of UEs first, and then decreases sharply and finally becomes stable. The performance peak occurs when the total number of UEs is equal to the number of receive antennas. The reason is mentioned in the previous paragraph. Moreover, a comparison between the proposed algorithm and the random power allocation algorithm is conducted. For the random power allocation algorithm, the transmit power follows a uniform distribution in the range of [0,26] dBm. And we calculate the mean value of 20 transmit power sets. It is observed that our proposed algorithm performs better than the random power allocation algorithm.

**Figure 5:**
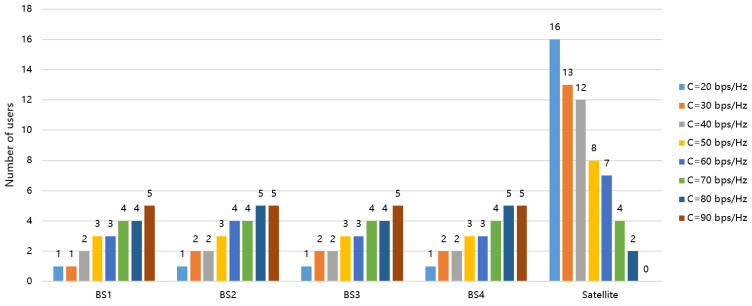
User association results.

In Figure 5, the user association results are presented for various sets of backhaul link rate *C*. The maximum transmit power is set as 26 dBm. It is seen that the distribution of the number of UEs accessing different terrestrial BSs is relatively even. As the backhaul link capacity increases, fewer UEs will access the satellite. This is because UEs will prioritize accessing terrestrial BSs with better channel conditions. However, in situations where the capacity of terrestrial BSs is constrained, UEs will turn to accessing the satellite in order to guarantee the fulfillment of their QoS requirements.

**Figure 6:**
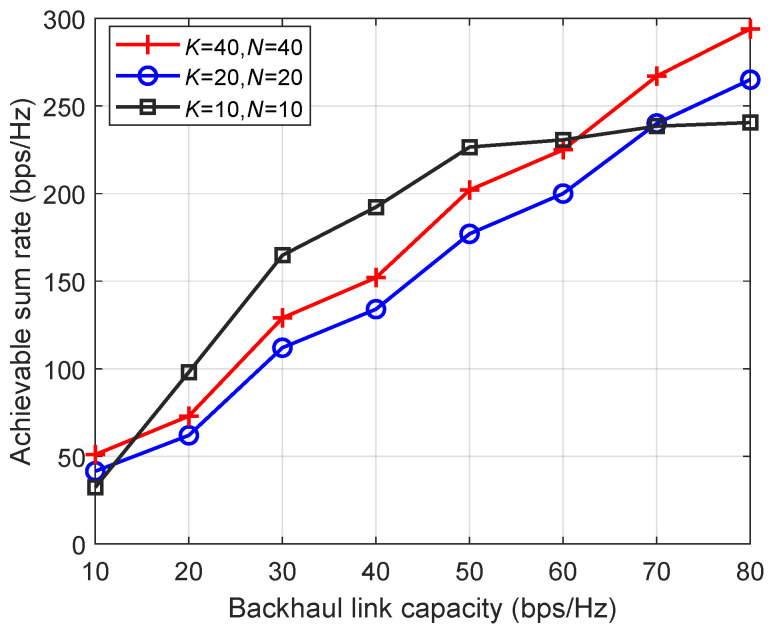
Achievable sum rate versus the backhaul link capacity.

Figure 6 presents the achievable sum rate versus the backhaul link capacity. The total number of UEs *K* and receive antennas *N* are set as follows: K=10, N=10, K=20, N=20, and K=40, N=40. The maximum transmit power is set as 26 dBm. It is seen that the performance increases with the increase in backhaul link capacity. When the backhaul link capacity is relatively low, the sum rate for 10 UEs is higher than the sum rates for 20 UEs and 40 UEs. This is because when the backhaul link capacity is small, the number of UEs that terrestrial BSs can accommodate is relatively limited, and excess UEs will access the satellite. This results in severe interference among the UE signals received by the satellite, thereby impairing the overall performance. When the number of UEs is relatively low, terrestrial BSs are sufficient to provide service to all UEs even with limited backhaul link capacity. As the backhaul link capacity of these BSs continues to rise, the achievable sum rate approaches saturation, leading to a flattened trend in the sum rate for 10 UEs thereafter.

## 5. Conclusions

This paper investigates a NOMA-based uplink ISTN, in which each user can access the satellite or terrestrial BSs according to their individual QoS requirements. In order to maximize the achievable sum rate, an optimization problem is established to jointly optimize the transmit power, user association, and receive beamforming. A two-stage algorithm is developed to solve the non-convex problem. At the first stage, user associations are determined based on individual preference lists and the backhaul capacities of the APs. At the second stage, the power allocation and the receiving beamforming vectors are alternately optimized in an iterative process. Within each iteration, the closed-form solution for the transmit power is derived. Simulation results validate the effectiveness of the proposed algorithm and the benefits brought by NOMA. UEs prefer to access terrestrial BSs when their backhaul link capacity is sufficient; otherwise, they can rely on the satellite for QoS guarantees. Furthermore, the achievable sum rate achieves its peak when the number of UEs aligns with the number of receive antennas at the APs.

## Figures and Tables

**Table 1:** Simulation parameters.

Parameter	Setting
Path loss model	$\beta {\left(\frac{d}{d_{0}}\right)}^{-\alpha }$ [15]
Parameters in path loss	β = −30 dB, $d_{0}$ = 1 m, α = 3, 2 [15]
Noise power	−174 dBm/Hz [15]
Altitude of satellite	300 km [1]
Number of BSs	*M* = 4
Coordinates of BSs	(0.75 km, 0.75 km), (0.75 km, 0.25 km)
	(0.75 km, 0.75 km), (0.75 km, 0.25 km)
Backhaul link capacity for BS	$C_{1}=C_{2}=\cdots =C_{M}=C$ = 50 bps/Hz
Number of receive antennas at BS	$N_{1}=N_{2}=\cdots =N_{M+1}=N$ = 10, 20, 40
Number of users	*K* = 10, 20, 30, 40

## Data Availability

Data are contained within the article. Further inquiries can be directed to the corresponding author.
